# Outcomes for group working: Contextualising group work within professionalism frameworks

**DOI:** 10.1111/medu.14781

**Published:** 2022-02-24

**Authors:** Kirsty R. McIntyre, Lynsay E. Crawford

## 1 WHAT PROBLEM WAS ADDRESSED?

Team working is a graduate attribute and professional requirement for medical students but can be difficult to teach and assess. A major challenge of group working at university is ensuring equal contribution to the group process (avoiding ‘free‐loaders’, i.e. non‐contributing peers^1^), and the fair allocation of marks commensurate with contribution.

At our institution, year 1 medical students must collaborate on data collection and preparation of a group presentation component (worth 25% of the overall grade) of a summative coursework task. Previously, we addressed the issue of ‘free‐loaders’ by asking all students to complete a Student Participation Agreement (SPA). The SPA was completed online and asked students to confirm (yes or no) whether all members of their group had contributed fairly to the project. Following review by the Assessment Team, individual students were awarded either the group mark in full or no award, resulting in a fail. This system identified group members who did not contribute at all but was not nuanced enough to identify students whose contributions did not meet peer expectations, for example, failed to complete tasks on time.

## 2 WHAT WAS TRIED?

We implemented peer assessment of individual contribution to the group task using an internally developed online system. The criteria chosen were aligned to GMC's Good Medical Practice, to set the task within a ‘graduate attribute’ framework and contextualise students' professionalism teaching. After the group presentation, students completed evaluations for each member of their group against five descriptors (attendance, shared responsibility, active participation, communication and respect) based on whether criteria were met ‘never (0)’, ‘sometimes (1)’ or ‘always (2)’. In line with other coursework there was a deadline to submit peer assessments. Students who marked their peers down were required to provide contextual feedback, which was moderated by staff for unduly harsh or inappropriate comments. Following staff review, the group grade was adjusted for 4 students who were marked down by several of their group. A further 4 students who did not complete peer assessment on time received standard late penalties.

## 3 WHAT LESSONS WERE LEARNED?

A survey of current year 1 (40/337, 12%) and year 2 (8/305, 3%) students, that is, students with experience of each system, was completed to evaluate the changes. Participants felt that the peer assessment system was a fair way of evaluating individual contribution to a group task (33/40, 83% of year 1 respondents agreed or strongly agreed versus 2/8, 25% of year 2 for the SPA). In contrast to the SPA, there were no student disputes with peer assessment because this model allows subtle grade adjustment and did not lead to any coursework fails. Students who were marked down by their peers were provided bespoke condensed feedback highlighting areas for improvement, thus supporting students in developing their professionalism and group working skills.

We have learned that students require reassurance that peer assessment is not punitive towards individuals with extenuating circumstances. Additionally, we found that some students held negative perceptions of group working and therefore have adopted the use of short videos to provide specific guidance and examples for students to model behaviour.

## References

[1] Hall D , Buzwell S . The problem of free‐riding in group projects: looking beyond social loafing as reason for non‐contribution. Active Learning in Higher Education. 2013;14(1):37‐49. doi:10.1177/1469787412467123

